# Characterization and Expression of the Zebrafish *qki* Paralogs

**DOI:** 10.1371/journal.pone.0146155

**Published:** 2016-01-04

**Authors:** Katarzyna J. Radomska, Jonathan Sager, Bryn Farnsworth, Åsa Tellgren-Roth, Giulia Tuveri, Christiane Peuckert, Petronella Kettunen, Elena Jazin, Lina S. Emilsson

**Affiliations:** 1 Department of Evolution and Development, Evolutionary Biology Centre, Uppsala University, Uppsala, Sweden; 2 Department of Neuroscience, Uppsala Biomedical Centre, Uppsala University, Uppsala, Sweden; 3 Institute of Neuroscience and Physiology, Department of Psychiatry and Neurochemistry, The Sahlgrenska Academy, University of Gothenburg, Gothenburg, Sweden; 4 Department of Neuropathology, Nuffield Department of Clinical Neurosciences, University of Oxford, John Radcliffe Hospital, Oxford, United Kingdom; The John Curtin School of Medical Research, AUSTRALIA

## Abstract

Quaking (QKI) is an RNA-binding protein involved in post-transcriptional mRNA processing. This gene is found to be associated with several human neurological disorders. Early expression of QKI proteins in the developing mouse neuroepithelium, together with neural tube defects in *Qk* mouse mutants, suggest the functional requirement of *Qk* for the establishment of the nervous system. As a knockout of *Qk* is embryonic lethal in mice, other model systems like the zebrafish could serve as a tool to study the developmental functions of *qki*. In the present study we sought to characterize the evolutionary relationship and spatiotemporal expression of *qkia*, *qki2*, and *qkib*; zebrafish homologs of human *QKI*. We found that *qkia* is an ancestral paralog of the single tetrapod *Qk* gene that was likely lost during the fin-to-limb transition. Conversely, *qkib* and *qki2* are orthologs, emerging at the root of the vertebrate and teleost lineage, respectively. Both *qki2* and *qkib*, but not *qkia*, were expressed in the progenitor domains of the central nervous system, similar to expression of the single gene in mice. Despite having partially overlapping expression domains, each gene has a unique expression pattern, suggesting that these genes have undergone subfunctionalization following duplication. Therefore, we suggest the zebrafish could be used to study the separate functions of *qki* genes during embryonic development.

## Introduction

Quaking (QKI) is an evolutionarily conserved RNA-binding protein involved in post-transcriptional mRNA processing, ranging from facilitating spliceosomal complex formation [1], to mRNA stability [2] and localization [3]. Bioinformatic analysis of the QKI response element (QRE) identified 1,433 putative RNA targets associated with processes such as development, organogenesis and cell differentiation [4]. Similar to other members of the Signal Transduction and Activation of RNA (STAR) protein family, QKI contains a single K-Homology (KH) motif directly involved in RNA binding [5]. The KH domain is expressed within each of the multiple isoforms of *QKI* (*QKI5*, *QKI6*, *QKI7*, and *QKI7b*), which are distinguished by both the size of the mRNA molecule and the C-terminal amino acid sequence [6]. In the human prefrontal cortex, *QKI* isoforms present differential expression patterns over time, with high *QKI5* expression in the fetal brain and an increased postnatal expression of *QKI6*, *7* and *7b* [7]. Alterations in the expression levels of specific *QKI* isoforms have also been associated with schizophrenia [8–11], major depression [12], anxiety [13], ataxia [14], and glioma [15–18], highlighting the importance of a better understanding of the diverse functions of *QKI*, particularly during nervous system development.

The function of *Qk* has predominantly been investigated in rodents where RNA coding for each isoform of *Qk* can be detected in most tissues of the adult mouse (Kondo et al., 1999). Within the nervous system, *Qk* is abundantly expressed in glial cells, including oligodendrocytes and astrocytes in the central nervous system (CNS), and Schwann cells in the peripheral nervous system (PNS), while it is absent from mature neurons, with the exception of transient expression of *Qk5* in motor neurons of the embryonic spinal cord [6, 19]. Similar to humans, expression of the mouse *Qk6* and *Qk7* isoforms peak during the active phase of myelination, while *Qk5* is upregulated during embryogenesis [6, 7, 20]. Consistent with their expression patterns, loss of QKI6 and QKI7 proteins specifically from myelin-forming glia (oligodendrocytes and Schwann cells) resulted in severe dysmyelination in both the CNS and PNS, in the original *Qk viable* (*Qk*^*v*^) mutant line, which carries a spontaneous deletion in the 5’ regulatory region of the gene. Consequently, *Qk*^v^/^v^ mice develop characteristic tremors (“quaking”) of the hindquarters around the second postnatal week, followed by tonic-clonic seizures in adults [6, 21–24]. A more severe dysmyelination phenotype, accompanied by early onset seizures and severe ataxia, was observed in mice carrying another viable *Qk* recessive allele [25]. This phenotype was attributed to the loss of *Qk6* and *Qk7*, similar to what has been observed in *Qk*^*v*^, but showing an additional reduction in the expression of the *Qk5* isoform. Finally, a complete knockout of *Qk* results in neural tube defects and mid-embryonic lethality [26], obfuscating functional characterization of this gene at later developmental timepoints.

In an attempt to identify an alternative model to study QKI functions during early vertebrate development *in vivo*, we investigated the homologs of *QKI* in zebrafish (*Danio rerio*). One of the zebrafish homologs, *qkia* (located on chromosome 17), has previously been cloned [27] and has been proposed to promote slow muscle formation by attenuating Hedgehog signaling through stabilization of *gli2a* (GLI family zinc finger 2a) mRNA [28]. However, two additional copies of the *qki* gene that have not been previously studied are present in the zebrafish genome, *qkib* (on chromosome 13) and *qki2* (on chromosome 12). Here we present the evolutionary relationship of the three zebrafish *QKI* homologs and describe their developmental expression, both spatially and temporally.

## Materials and Methods

### Sequences retrieval and exon-intron organization

Nucleotide and amino acid (aa) sequences of human and zebrafish *Quaking* were retrieved from Genbank (version 197). The accession numbers of the selected sequences are: QKI5 (NP_006766.1), QKI6 (NP_996735.1), QKI7 (NP_996736.1) and QKI7B (NP_996737.1), Qkia_tv1 (NP_571299.1), Qkia_tv2 (XP_005158811.1), Qki2_tv1 (XP_005156410.1), Qki2_tv2 (NP_957136.2), Qkib_tv1 (XP_005156558.1), Qkib_tv2 (XP_003199734.1) and Qkib_tv4 (XP_005156556.1). Genbank sequences were compared against the Ensembl assembly Zv9 (release 70) to verify their presence in the genome. In addition, Qki2_tv3 and Qkib_tv3 were manually retrieved from Ensembl using BLAST with QKI5 as the query. The Qkib_tv1 (XP_005156558.1) was modified by the addition of exon 8. To obtain the exon-intron structure of *qki* in other species, and to identify additional putative transcript variants in zebrafish, the four human QKI aa sequences listed above were used for a BLAST query against the genomes of *Branchiostoma floridae* (amphioxus), *Danio rerio* (zebrafish), *Lepisosteus oculatus* (spotted gar), *Latimeria chalumnae* (coelocanth), and *Gallus gallus* (chicken). This analysis identified the translated exon sequences from each genome and the aligned human exons were used to manually annotate the splice sites in each species (listed in S1 Fig). Small exons not detected by BLAST were identified using six-frame translation of the relevant genomic sequence.

### Bioinformatic analysis

Sequences were aligned using Clustal Omega 1.2.0 [29]. The resulting aa alignment and corresponding DNA sequences were loaded in PAL2NAL, v14 [30] to construct a codon alignment. Manual editing of alignments was done in JalView, v2.8.0b1 [31] and the resulting alignment was used to assess the nucleotide substitution model with jModelTest 2.1.4 [32, 33]. A phylogenetic tree was constructed with MrBayes 3.2.1 [34], using the standard nucleotide substitution model, allowing all rate to vary as predicted in the general time reversible model, and assuming a gamma distribution of rates with invariable sites. The analysis was run for 1,000,000 generations with a sample frequency of 100 and a relative burnin of 25%.

For synteny analysis between human and zebrafish chromosomes containing the *QKI*/*qki* genes, two different methods were employed. For an analysis of larger chromosome regions, the Synteny Database (http://syntenydb.uoregon.edu/synteny_db/) [35] was used, and microsynteny was investigated manually with Ensembl.

### Zebrafish

Zebrafish AB strain embryos were raised in system water at 28.5°C and staged according to either hours post-fertilization (hpf) or morphological criteria [36] as indicated in the text. For *in situ* hybridization experiments, embryos older than 24 hpf were treated with 0.003% 1-phenyl 2-thiourea (Sigma) to inhibit pigmentation. All animal studies were carried out with approval from the animal ethics committee (Uppsala djurförsöksetiska nämnd) in Uppsala, Sweden, permit number: C262/11 and C161/14.

### RNA extraction and real-time RT-PCR

Total RNA was extracted from pooled embryos or larvae using Trizol (Life Technologies) according to the manufacturer’s protocol. RNA concentration and quality were determined using a NanoDrop ND-1000 spectrophotometer. From each biological replicate, 400 ng of RNA were reverse transcribed into cDNA using TaqMan Reverse Transcription Reagents (Applied Biosystems) according to manufacturer's protocol. cDNA samples were diluted with RNAse-free water to a final concentration of 5 ng/μl and stored at -20°C.

All real-time PCR experiments were performed using an ABI Prism 7500 Sequence Detector System (Applied Biosystems) with optical tubes (Applied Biosystems). Amplification was achieved using Power SYBR Green PCR Master Mix (Applied Biosystems), equal amounts of cDNA, and gene specific primers sets: *qkia*, *5’-* CCCACTGGAGTTACCAGAGC, 5’- CAGTTGCTTCGCTGTGAGTC; *qki2*, 5’- CCAGAGTCCGGCATCATCTA, 5’- TTTTGTCGGGAAAGCCATAC; *qkib*, 5’- TGGAGTATCCCATCGACTCC, 5’- TGGGAATGTGACAGGTCTGA. Following completion of each real-time PCR reaction, a dissociation step was added and melt curve analysis was performed to validate the specificity of PCR amplicons. Data were processed by ABI 7500 system SDS software version 2.0.6. Relative *qki* mRNA levels were quantified using a standard curve and normalized against expression of two endogenous control genes previously validated as suitable reference genes for developmental studies in zebrafish [37]: *eukaryotic translation elongation factor 1 alpha 1*, *like 1 (eef1a1l1)*: 5’- AGCAGCAGCTGAGGAGTGAT, 5’- CCGCATTTGTAGATCAGATGG, and *actin*, *beta 1 (actb1)*: *5’-* GATGATGAAATTGCCGCACTG, 5’- ACCAACCATGACACCCTGATGT.

### cRNA probe synthesis and whole mount *in situ* hybridization

Templates for probe synthesis were PCR amplified from embryonic zebrafish cDNA using primers including SP6 or T7 RNA polymerase promoter sequences. To minimize cross-reactivity, the 5' untranslated regions of *qkib* and *qki2* were used for primer design. Primer sets were designed as follows: *qkia*, 5’-CTGTAATACGACTCACTATAGGGCGTAATGAACACAGAGAAAC, 5’- GGGATTTAGGTGACACTATAGAAACACCCAGTTTAAGAGAAAG; *qki2*, 5’- CTGTAATACGACTCACTATAGGGGCAGAGAACAGCACTGAACAC, 5’- GGGATTTAGGTGACACTATAGAAATGTTTGTTGGATGTTTGACG; *qkib*, 5’- CTGTAATACGACTCACTATAGGGAGACTTGGCTTCCCTCTTCAC, 5’- CTGTAATACGACTCACTATAGGGAGACTTGGCTTCCCTCTTCAC. All PCR products were of expected size as inspected by agarose gel electrophoresis. Purified PCR products were *in vitro* transcribed and labeled using digoxigenin (DIG) RNA Labeling Kit (Roche) according to manufacturer’s protocol. cRNA probes were precipitated with LiCl and stored at -80°C. Whole mount *in-situ* hybridization was performed according to a standard protocol [38]. Between 100 and 250 ng of each probe was hybridized overnight at 67°C.

### Fluorescent *in situ* hybridization and immunofluorescence

Embryos were fixed in 4% paraformaldehyde (PFA) in PBS at specified times post-fertilization, cryoprotected in 30% sucrose, and sectioned at 12 μm using a Leica CM3050 S cryostat. Sections were permeabilized with 2.5 μg/ml Proteinase K for 2 minutes at room temperature, and post-fixed in 4% PFA/PBS for 15 minutes. Sections were then treated with 0.1 M triethanolamine/ 0.25% acetic anhydride for 10 minutes and incubated with either 150 ng of DIG or FITC-labeled probe per slide overnight at 65°C. Probe was detected with an anti-DIG or anti-FITC antibody (1:100) conjugated to horseradish peroxidase (Roche). Signal was produced by treatment with TSA Plus Cyanine 3 / Cyanine 5 system (PerkinElmer) and washed overnight at 4°C. Immunofluoresence was completed using anti-HuC/D (6 μg/ml, Life Technologies), anti-SOX2 (3 μg/ml,Abcam), and primary antibodies were detected using an anti-rabbit antibody conjugated to Alexa-488 (Abcam) and anti-mouse antibody conjugated to Alexa-594 (Life Technologies). DAPI was added at a concentration of 1 μg/ml.

### Imaging

Zebrafish embryos for whole mount *in situ* hybridization were embedded in 75% glycerol/PBS and imaged using a Nikon SMZ 1500 microscope equipped with a DS-Vi1 camera. Fluorescent images of transverse cryostat sections were acquired using a Leica TCS SP5 laser-scanning confocal microscope.

## Results

### *qki* phylogeny in chordates

**Fig 1:**
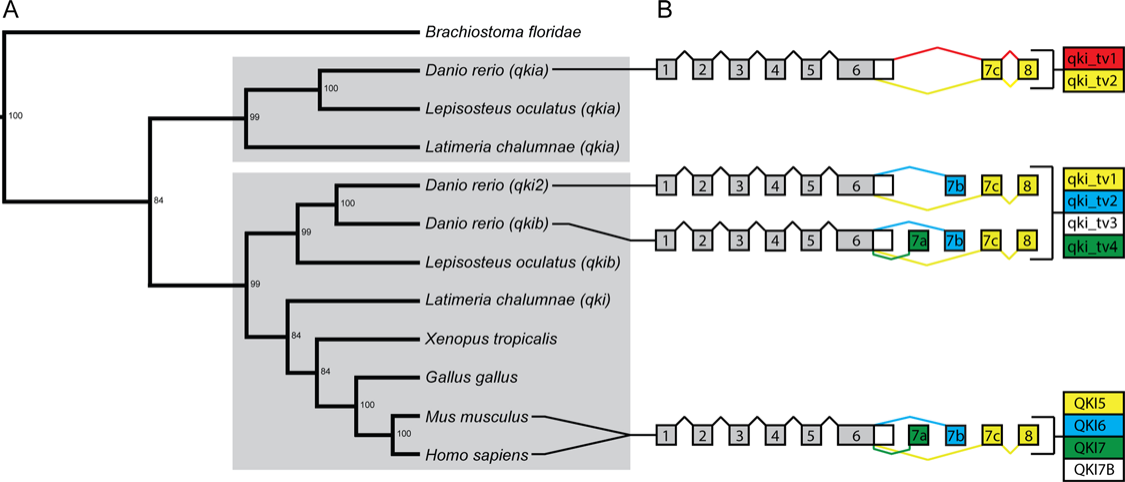
Phylogeny and genomic organisation of *qki* genes. **(A)** Phylogenetic relationships of chordate *qki* genes inferred from Bayesian analysis of human *QKI5* nucleotide sequence and corresponding transcripts from selected species. In the case of multiple genes in one species, each copy is identified by its Ensembl gene name. Nodes are labeled with posterior probability values. The two main branches are denoted by grey boxes. **(B)** Exon-intron structure of predicted *qki* transcript variants in zebrafish and mammals. Exons are shown as boxes and introns as lines. The human exon terminology is used for all species, numbering the exons 1–6, 7a, 7b, 7c and 8. The exons are not drawn to scale, the correct sizes are presented in S1 Fig. The colors of the 3’ exons and introns match the transcript variants that include them. The names of the transcript variant are indicated in the boxes to the right, and the colors are according to their similarity with the human transcript variants. The white boxes indicate transcripts containing an extended version of exon 6.

As a first point of investigation of the *qki* genes in zebrafish, we examined the evolution of *qki* genes during chordate radiation. We identified three zebrafish genes encoding for proteins with high amino acid similarity to the single human *QKI* gene (> 75%). BLAST searches also revealed that the human *QKI5* isoform had annotated transcript structures similar to those in the investigated species. These *QKI5*-related sequences were used to construct a phylogenetic tree, using the single *qki* gene in amphioxus as an outgroup (Fig 1A). This analysis revealed that the zebrafish *qkia* gene diverged early in vertebrate evolution, forming a separate clade with one of the two *qki* genes present in both *L*. *oculatus* (spotted gar) and *L*. *chalumnae* (coelocanth). The other two identified zebrafish *qki* genes, *qki2* and *qkib*, cluster with the second *qki* gene present both in spotted gar and coelocanth, along with the single *qki* gene identified in tetrapods, a selection of which is shown in the phylogenetic tree (Fig 1A). This topology suggests that the zebrafish *qki2* and *qkib* are orthologs of human *QKI*, while the zebrafish *qkia* gene is a paralog of *qki2* and *qkib* in the zebrafish and of *QKI* in humans.

### Exon-intron structure of human and zebrafish *QKI* gene transcripts

To examine the conservation of gene structure between zebrafish *qki* genes and human *QKI* we compared the exon-intron structure of the different predicted transcript variants (tv) of the zebrafish against the human transcripts (Fig 1B). The human exon terminology is used for zebrafish, numbering the exons 1–6, 7a, 7b, 7c and 8. Note that the numbering is based on exon structure similarity. The exon size is generally well conserved with exceptions in exons 1, 6, and 7a (S1 Fig).

Exon-intron structure of predicted *qki* transcript variants presents additional evidence of the relationships between the three zebrafish *qki* genes and human *QKI* (Fig 1B). The *qkia* in zebrafish includes two transcript variants that differ only in the extension of exon 6, as previously shown by Lobbardi et al. 2011. The first transcript variant, *qkia_tv1*, includes an extended sequence of exon 6 together with exon 7c and 8. This combination of exons is not found in any other gene analyzed in this study. The second transcript, *qkia_tv2*, shares identical exon structure with human *QKI5* (marked in yellow). Three transcript variants were predicted for *qki2*, among which *qki2_tv1* and *qki2_tv2* have structure similar to human *QKI5* and *QKI6*, respectively. It should be noted that the third *qki2* predicted transcript, *qki2_tv3*, is not annotated in either the Genbank or the Vertebrate genome annotation (Vega) databases. Furthermore, the extended versions of exon 6 shares reduced sequence similarity between humans and zebrafish. Therefore, the C-terminus of *qki2_tv3* transcript, if produced, is not conserved with human *QKI7B*. The third zebrafish *qki* gene, *qkib*, encodes four predicted transcripts, where the first three share the same structure with the three transcripts identified for *qki2*. The fourth predicted transcript shares a similar structure with human *QKI7*.

### Shared synteny between zebrafish and human *QKI* genes

**Fig 2:**
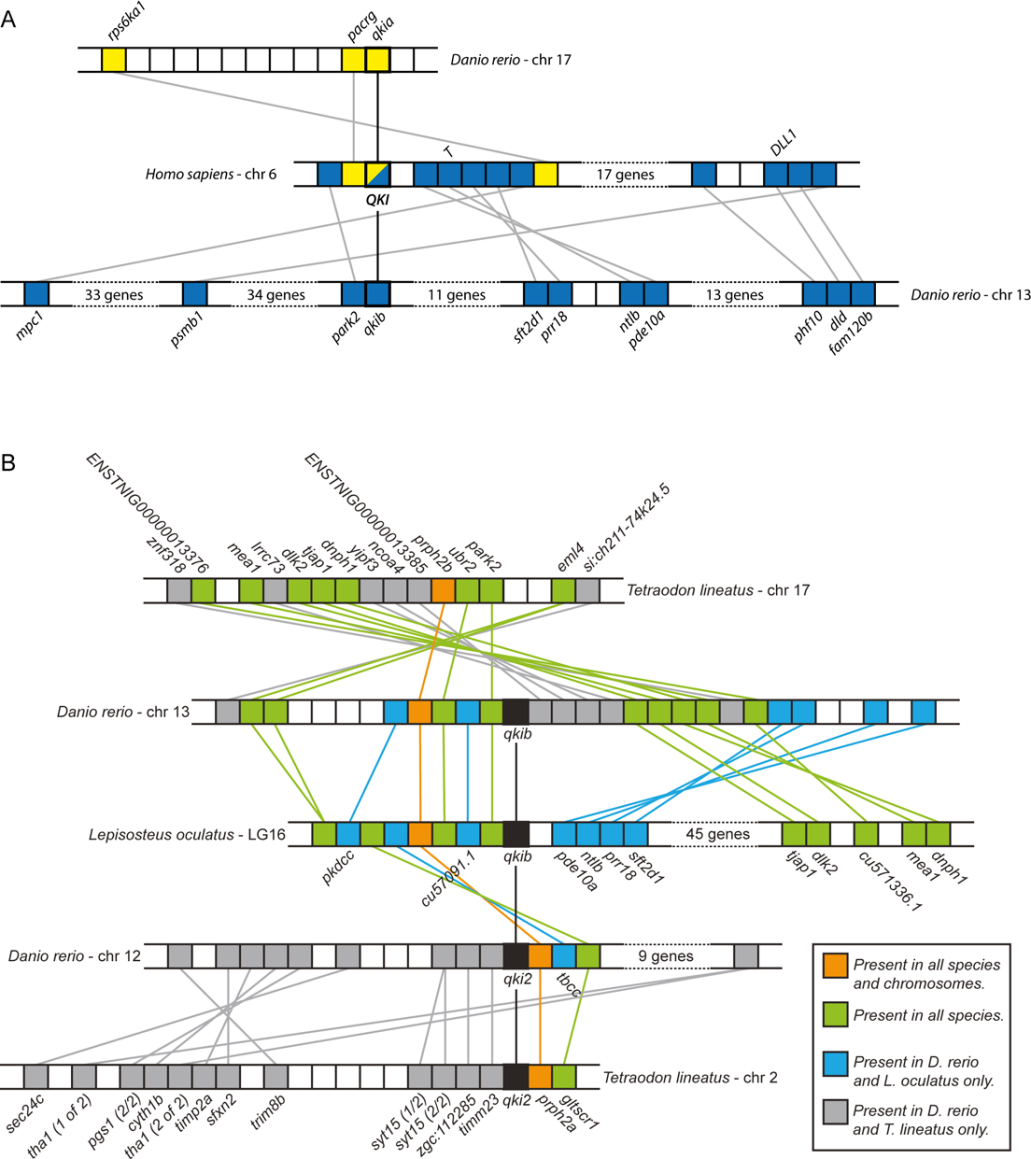
Syntenic conservation of *qki* genes. **(A)** Shared synteny between the genomic region containing *QKI* in human chromosome 6 and zebrafish chromosomes 13 and 17. Colored boxes represent genes. Genes in common between human chromosome 6 and zebrafish chromosome 17 are marked in yellow and genes shared between human chromosome 6 and zebrafish chromosome 13 are colored blue. *QKI* is framed with a thick line. Gene names are the same in zebrafish and human unless marked otherwise. The chromosomes are not shown to scale. **(B)** Shared synteny between spotted gar (*Lepisosteus oculatus*) linkage group (LG) 16, zebrafish (*Danio rerio*) chromosomes 13 and 12, and Tetraodon (*Tetraodon lineatus*) chromosomes 17 and 2. Black boxes indicate *qki* homologs and colors represent conservation between species as described in the legend.

Given the high similarity between the *qki* genes, we investigated the synteny of these genes in multiple vertebrate species. Overall, a large degree of rearrangements were observed between all three zebrafish chromosome regions compared to the human region (Fig 2A). However, both *qkib* and *qkia* in the zebrafish share multiple genes, on a macrosyntenic level, with human *QKI*. The chromosome containing *qki2* did not share any synteny with the human *QKI* genomic region, and was therefore not included in the figure. However, the *qki2* genomic region of zebrafish shares a large degree of synteny with other teleost fish species, such as the region surrounding *qki2* of the tetraodon and *qkib* of the spotted gar (Fig 2B). Conversely, analysis of the same region anchored to zebrafish *qkib* shows a relatively increased synteny with the *qkib* gene of spotted gar. This suggests that *qki2* is a teleost-specific gene that likely originated from the whole genome duplication at the root of the teleost lineage.

### Protein alignments between zebrafish and human QKI

**Fig 3:**
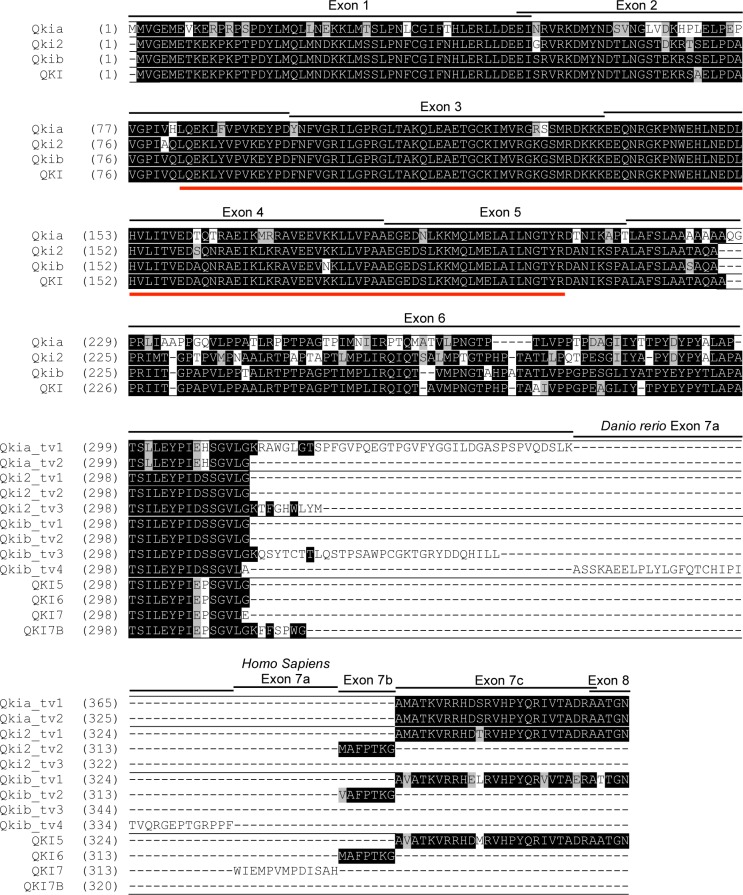
Sequence alignment and conservation between zebrafish and human QKI proteins. Amino acid sequence alignment including all isoforms found in human and zebrafish. The position of each exon is indicated with black lines above the alignment. The human exon terminology is used for both species. The KH domain is marked with a red line below the alignment. Black background indicates identical amino acids and grey indicates conservative changes.

The alignment of all translated and predicted zebrafish and human QKI protein isoforms demonstrates high amino acid (aa) conservation within the first five exons encoding for the functional KH domain (Fig 3). Highest similarity was found between zebrafish Qkib and human QKI, with only two aa substitutions located in exons 2 and 4. Qki2 differs in six positions with the human protein while Qkia is the most divergent zebrafish Qki isoform in this region. In exon 6, larger amounts of aa substitutions are present between the two species, with the highest conservation still present between human and Qkib. The extended version of exon 6, only present in one protein isoform encoded by each gene in both species, is highly dissimilar. The same is true for exon 7a included in the sequence of Qkib_tv4 and QKI7. Within most C-terminal exons, both isoforms of Qkia align with QKI5, while the isoforms of Qki2 and Qkib align either with QKI5 or QKI6.

### Developmental expression profiles of *qki* genes in zebrafish

**Fig 4:**
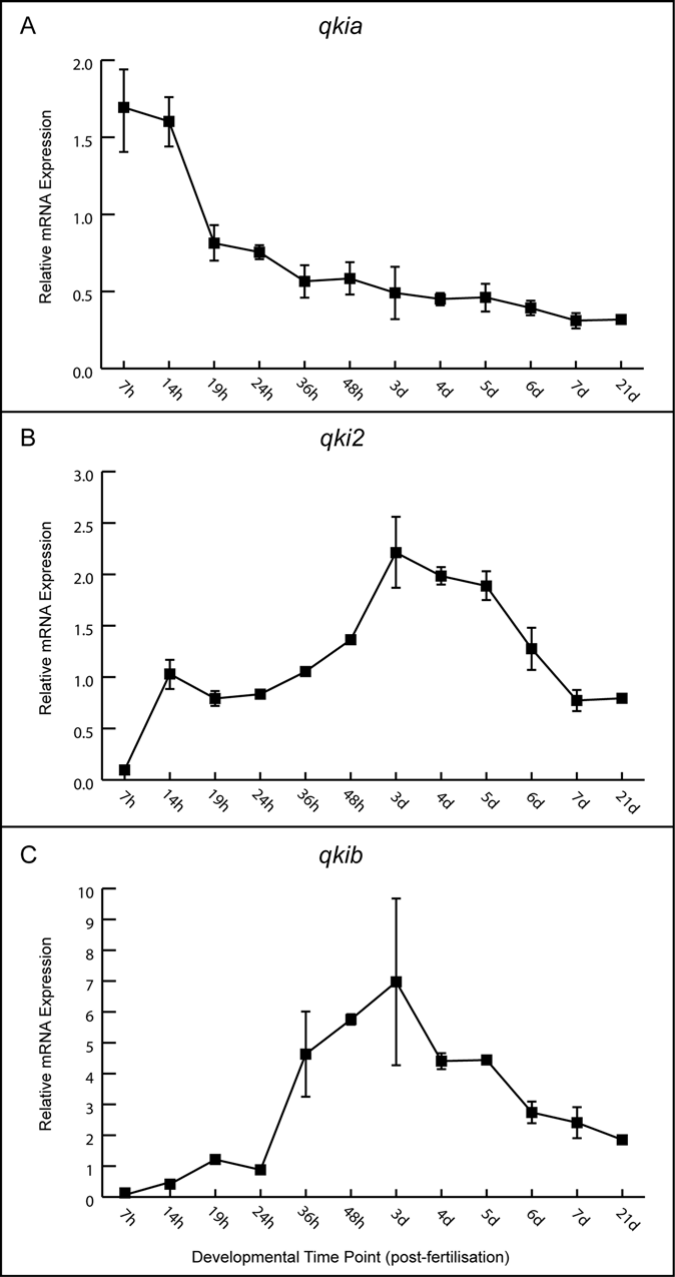
Quantitative temporal expression of *qki* transcripts during development. Relative mRNA expression of *qkia*, *qki2*, and *qkib* is plotted across indicated developmental time points. Each point represents the average measurement of three biological replicates. Bars correspond to standard deviation of the mean. hpf: hours post-fertilization, dpf: days post-fertilization.

Developmental gene expression profiles of *qkia*, *qki2* and *qkib* were determined using quantitative real-time PCR (qPCR) on RNA extracted from whole zebrafish embryos and larvae, ranging from 7 hours post-fertilization (hpf) to 21 days post-fertilization (dpf) (Fig 4). Primers used were designed to include all potential transcript variants for each gene. The *qkia* gene showed the highest expression during early embryogenesis (7–14 hpf) and its transcription rapidly declined at the end of somitogenesis (19 hpf) by approximately 50% (Fig 4A). Over the following 6 days of development *qkia* expression gradually decreased to about 25% of the initial transcript level, which remained stable until 21 dpf. The relative mRNA levels of *qki2* and *qkib*, while distinct from *qkia*, followed similar developmental patterns, including up-regulation during early development (between 7–14 hpf for *qki2* and 24–36 hpf for *qkib*). Expression of both *qki2* and *qkib* peaked at 3 dpf, and gradually declined to about 30% of the maximum level by 7 dpf, which remained stable until 21 dpf (Fig 4B and 4C).

**Fig 5:**
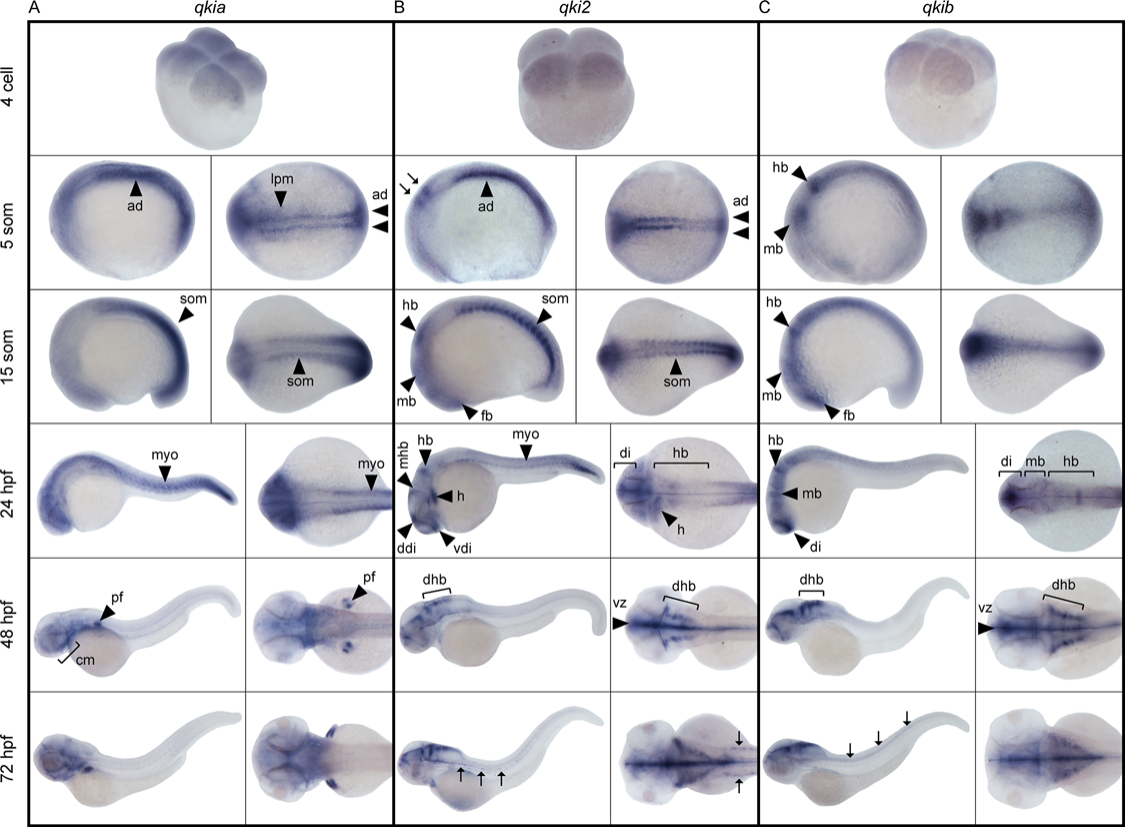
Spatiotemporal expression of *qki* genes during development. Representative images of whole mount *in situ* hybridization using probes detecting *qkia* **(A)**, *qki2* **(B)** and *qkib* **(C)** expression. Developmental stages are expressed as the number of cells, somites (som), or hours post-fertilization (hpf). Lateral (left panels) and dorsal (right panels) views are shown, anterior to the left. Arrows in **B** at the 5 somite stage indicate the hindbrain primordium, while arrows at 72 hpf indicate the presumptive lateral line Schwann cells and in **C**, developing trunk neural tube. ad: adaxial cells, lpm: lateral paraxial mesoderm, som: somites, fb: forebrain, di: diencephalon, vdi: ventral diencephalon, ddi: dorsal diencephalon, dhb: dorsolateral hindbrain, mb: midbrain, mhb: midbrain/hindbrain boundary, hb: hindbrain, vz: ventricular zone, myo: myotome, pf: pectoral fin, cm: cranial muscles, h: heart.

To compare the spatiotemporal expression of mRNAs encoding each of the three *qki* genes, zebrafish of various developmental stages were subjected to whole mount *in situ* hybridization using gene-specific cRNA probes (Fig 5). At the 4-cell stage, maternally transcribed *qkia* was detected, while no obvious staining was observed for either *qki2* or *qkib* probes, even after prolonged incubation with an alkaline phosphatase substrate. During early somitogenesis (5 somites), the *qkia* probe homogeneously labeled a subset of the presomitic mesoderm, named adaxial cells (ad), located in two rows tightly flanking the notochord (Fig 5A). Weaker and more diffuse staining was also detected in the lateral portion of paraxial mesoderm (lpm) as well as in the mesoderm of the head region. Expression of *qki2* mRNA was also found in the adaxial cells, however it was upregulated in newly formed somites (som) as compared to the presomitic paraxial mesoderm (Fig 5B). Different from *qkia*, *qki2* expression was also evident in two discrete stripes in the hindbrain primordium (arrows in Fig 5B, 5 somite stage). Transcription of *qkib* was detected in two clusters likely corresponding to the midbrain (mb) and hindbrain (hb) (indicated by arrows in Fig 5C, 5 somite stage), and in the neural plate of the trunk. As somites matured, the territories of both *qkia* and *qki2* transcription expanded laterally within the somite, with *qkia* expansion being more pronounced in the posterior segmental plate (Fig 5A and 5B). At the same time, *qki2* showed a similar pattern to *qkib* within the developing neural tube, where both transcripts were present in newly formed neuromeres, including forebrain (fb), midbrain and hindbrain (Fig 5B and 5C). However, *qkib* mRNA was more ubiquitously expressed within the developing neural tube and extended along the anterior-posterior axis.

By 24 hpf, the somite formation was complete and differentiated muscle fibers were labeled with both *qkia* and *qki2* probes (Fig 5A and 5B). Furthermore, *qkia* was diffusely expressed in the head, potentially labeling the craniofacial musculature, which became more apparent at later stages. On the other hand, *qki2* was strongly expressed in the heart primordium (h). Both *qkib* and *qki2*, but not *qkia*, were clearly expressed in the developing brain. The *qki2* staining was apparent in discrete regions, including the ventral and dorsal diencephalon (di), midbrain-hindbrain boundary (mhb) and hindbrain rhombomeres. Although *qkib* labeling was broadly expressed along the entire length of the neural tube (indicated by arrows in Fig 5C, 72 hpf), a more intense signal was detected in the diencephalon, midbrain and hindbrain.

During the hatching period (48–72 hpf), *qkia* was strongly expressed in the craniofacial muscles (cm) supporting the extending jaw and eye capsule. Labeling was also prominent in pectoral fin muscles (pf). However, neither *qkia* nor *qki2* remained transcribed in skeletal trunk muscles. Instead, *qki2* continued to be expressed in the heart and brain, where previously broader expression domains became progressively restricted to the ventricular zone (vz) across the developing brain. Moreover, two columns of *qki2*-positive cells could be distinguished in the dorsolateral hindbrain (dhb). From 2 to 3 dpf the pattern of *qkib* transcription in the brain was similar to *qki2* expression (Fig 5C). Additionally, at 3 dpf *qki2*, but not *qkib*, was detected along the anterior lateral line (indicated by arrows in Fig 5B, 72 hpf), suggesting *qki2* is present in Schwann cells myelinating the lateral line nerve. As the myelination progressed, *qki2* territory also expanded caudally along the entire length of the lateral line (not shown), consistent with the anterior-posterior maturation gradient of the trunk.

Together, the detected gene expression patterns suggest the possibility of both complementary and distinct functions of the *qki* genes during zebrafish development.

### *qki2* and *qkib* expression in the developing nervous system

**Fig 6:**
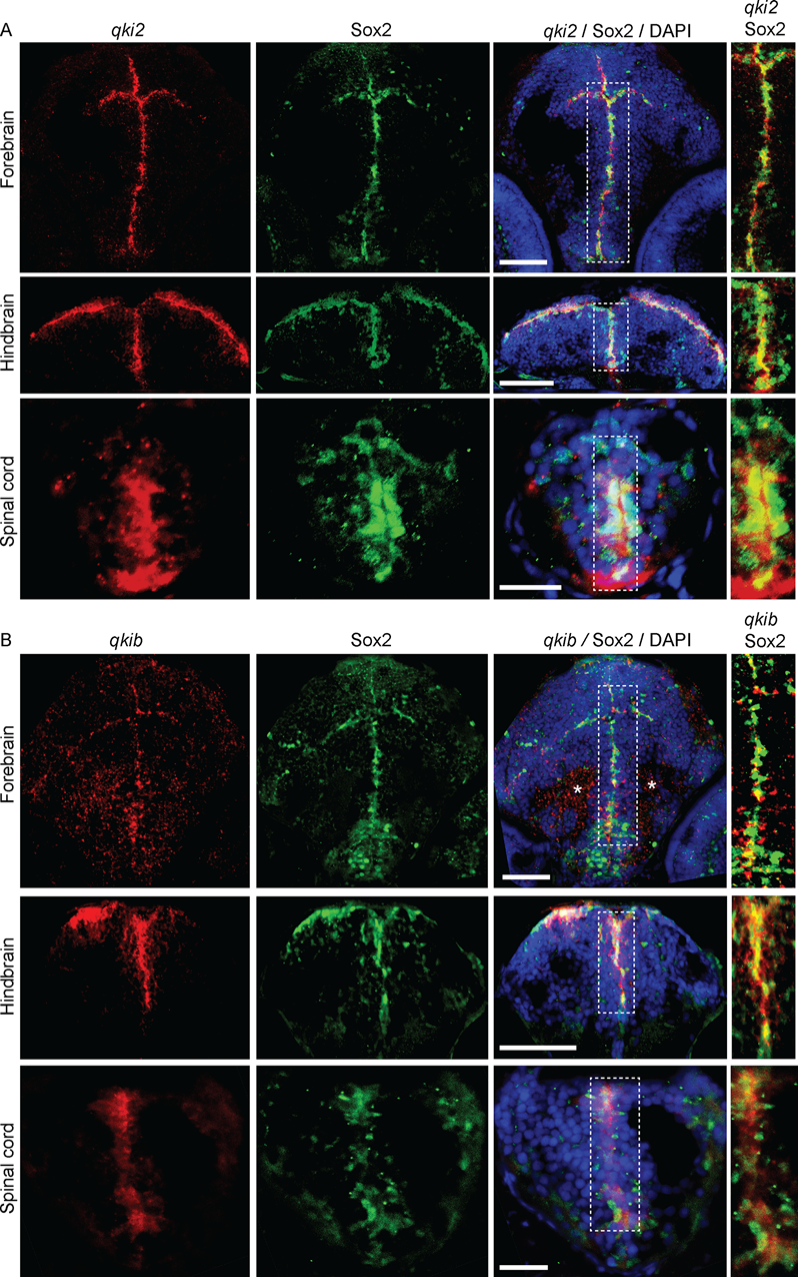
*qki2* and *qkib* are expressed in neural progenitor cells. Representative images of combined *in situ* hybridization and immunofluorescence detecting *qki2* **(A)** and *qkib* probes **(B)**, both shown in red. Sox2 antibody is shown in green and DAPI in blue. Images shown are within the forebrain, hindbrain and spinal cord and overlays are denoted in the figure header. Scale bars are 50μm for forebrain and hindbrain images, and 20μm for spinal cord images. Asterisks indicate staining artefacts.

**Fig 7:**
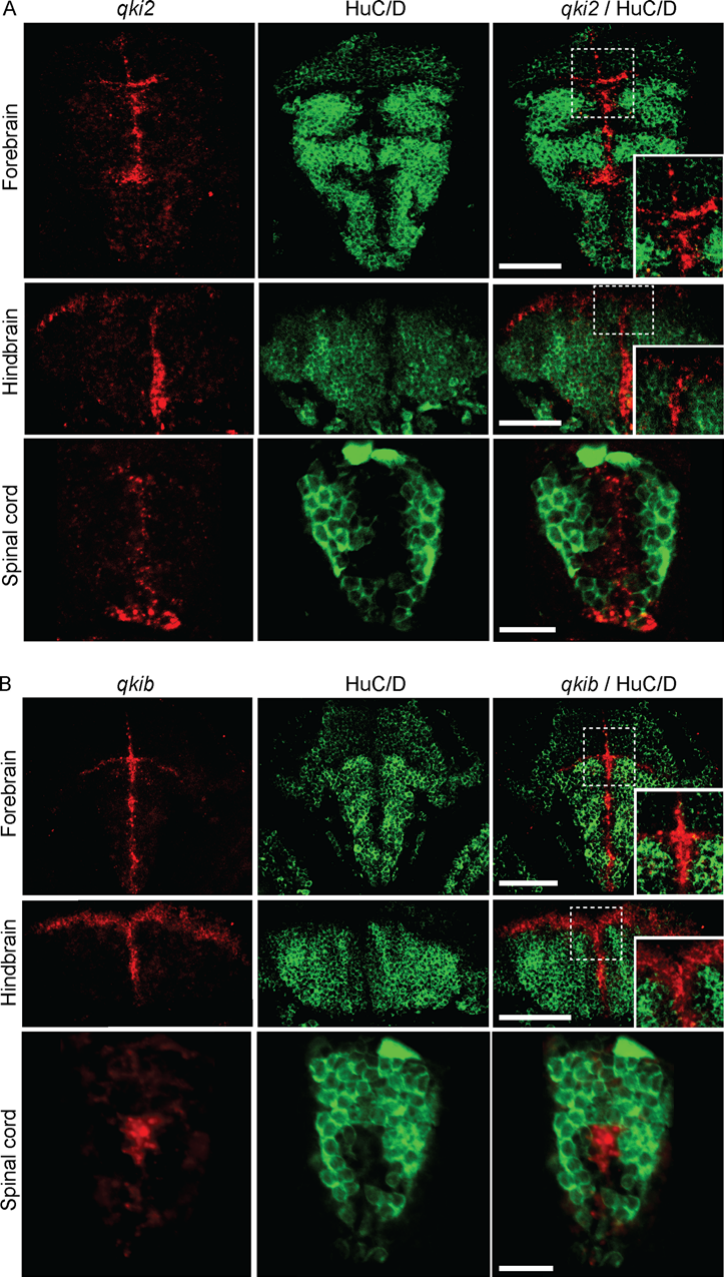
*qki2* and *qkib* are absent from differentiated neurons in developing zebrafish. Representative images of combined *in situ* hybridization and immunofluorescence detecting *qki2* (A) and *qkib* probes **(B)**, both shown in red. HuC/D antibody is shown in green. Images shown are within the forebrain, hindbrain and spinal cord.

We next sought to further examine the expression of *qki2* and *qkib* in the developing nervous system using *in situ* hybridization combined with immunofluorescence on transverse sections of developing zebrafish (Figs 6 and 7). At 3 dpf, both *qki2* and *qkib* were localized to the midline ventricular zone of the forebrain, visualized by co-labeling with Sox2, a marker of neural progenitor cell populations (Fig 6).

In the hindbrain, *qki2* and *qkib* were expressed in cells located in the dorsolateral region overlapping with Sox2 expression (Fig 6). Within the spinal cord, signal for both *qki2* and *qkib* was detected in the central progenitor zone at 2 dpf and 3 dpf, respectively, as shown by co-localization with Sox2 (Figs 6 and 7). At 3 dpf *qki2* expression was also detected in a ventral region, likely corresponding to the ventral floor plate of the developing spinal cord, outside of the labeled Sox2 progenitor zone (Fig 6). Notably both *qki2* and *qkib* were absent from differentiated neurons, demonstrated by lack of co-staining with an anti-HuC/D antibody (Fig 7). In summary qki2 and qkib are predominantly expressed in neural progenitor cells but not in differentiated neurons.

## Discussion

In this study, we investigated the evolution and expression of three *qki* genes in zebrafish: *qki2* and *qkib* which are orthologs of human *QKI* and *qkia* which is a paralog. Expression of *qkia* was primarily restricted to the mesoderm, whereas *qkib* mRNA was detected in the developing nervous system and *qki2* had a partially overlapping expression with both genes. Within the nervous system, *qki2* and *qkib* were both expressed in midline progenitor zones, and colocalized with Sox2, a marker of neural progenitor cells.

To investigate the evolution of *qki* genes, phylogenetic analysis was performed and revealed the segregation of two *qki* clades in vertebrates. Because only a single gene was identified in amphioxus, it is likely that two *qki* genes existed in the last common ancestor of tetrapods and ray-finned fish, a hypothesis supported by the presence of two distinct *qki* genes in both the spotted gar and coelocanth. The absence of two *qki* genes in the examined tetrapod genomes suggests that one of the ancestral paralogs was lost during the fin-to-limb transition. The gene *qki2* is likely the result of the teleost-specific whole genome duplication. This duplication event is supported by the increased syntenic conservation of the *qki2* genes in tetraodon and zebrafish, but not spotted gar. Furthermore, the human *QKI* locus shared neighboring genes with both *qkia* and *qkib*, but not *qki2*, supporting the teleost-specific origin of *qki2*. Similar conclusions were obtained by alignment of the human splice variants against the zebrafish genome; both *qki2* and *qkib* included putative transcripts with similar structures to human *QKI5* and *QKI6*, whereas of the two putative *qkia* transcripts, only one shared a similar transcript structure to *QKI*. At the amino acid level, there is a striking degree of conservation among all chordate Qki proteins throughout the RNA-binding KH-domain, suggesting a strong selective pressure on the composition of this domain. The C-terminus of QKI5 has previously been shown to be critical in the nuclear localization of this isoform [39], and sequence similarity between all three zebrafish isoforms (Qkia_tv1 and 2, Qki2_tv1 and Qkib_tv1) is highly conserved with human QKI5, suggesting that a nuclear localization of Qki is an ancestral trait. Similarly, the C-terminus of QKI6, which is capable of binding the TGE (*tra-2* and *GLI*) translation regulation element [40], is well conserved with both Qki2_tv2 and Qkib_tv2, suggesting that this function of the gene arose before the teleost-specific *qki* duplication. More divergence is apparent in the C-terminus of the QKI7 and QKI7B isoforms, suggesting that these isoforms are the result of more evolutionarily recent isoform acquisition.

Gene expression analysis of the *qki* genes in zebrafish demonstrated that the mRNA profiles of the three zebrafish *qki* genes exhibit unique, although partially overlapping expression patterns during embryonic and larval development. This is in contrast to the widespread expression of the single mammalian *QKI* gene in the heart, vascular system, muscles, brain and spinal cord [15, 20, 41]. The differences in expression between fish and mammals suggest that following the loss of *qkia* in the tetrapod lineage, the enhancer elements for *QKI* became more generalized, driving expression in both mesoderm and neural ectoderm lineages. Conversely, following the duplication events in the teleost, the *qki2* and *qkib* promoters diverged, resulting in retention of the two paralogs.

In accordance with a previous publication [27], *qkia* was detected in the trunk paraxial mesoderm and its derivatives, including somites and somite muscles as well as in the craniofacial and pectoral fin musculature. However, the strong *qkia* staining in the brain observed by Tanaka et al. could not be reproduced in our experiments.

*qkib* was detected in the neural plate during early somitogenesis with subsequent expression in the developing neural tube, where initially broad expression domains became progressively restricted to the CNS ventricular zone across brain and spinal cord. This spatiotemporal profile is indicative of *qkib* expression in neural progenitor cells, supported by both colocalization with Sox2, and a lack of *qkib* mRNA differentiated neurons, consistent with patterns previously reported for QKI isoforms in the mouse [6]. The function of *qkib* in this area is not yet known.

*qki2* was detected in partially overlapping expression domains with *qkia* within the paraxial mesoderm and somite muscles, and *qkib* within the developing neural tube. Additionally, in the nervous system, *qki2* exhibited unique expression patterns to *qkib*. The localization of *qki2* in the ventral spinal cord and upregulation of *qki2* transcription during the active phase of myelination (3 dpf), suggests that *qki2* may play a role in zebrafish myelination [42], similar to what has been found in the mouse [43]. However, additional cellular markers will be needed to further delineate the exact cellular expression of *qkib* and *qki2* within the nervous system.

Taken together, the distinct spatiotemporal localizations of the *qki* genes in zebrafish suggest that following duplication these paralogs have undergone subfunctionalization by mutations in their enhancer elements. Therefore, the zebrafish presents a unique model system with the potential to selectively dissect the role of *qki* in specific cell types. Future efforts will utilize directed genomic inactivation using gene editing techniques.

## Supporting Information

S1 Fig Genomic organization of human *QKI* and zebrafish *qki*. Known and predicted splice variants of the human *QKI* and the three zebrafish *qki* genes are detailed. The splice acceptor (sa) and splice donor (sd) sites for each exon and the exon length (in nucleotides) are compared between species.
(TIF)
