# Supplementary figures and images for: Characterization and Expression of the Zebrafish qki Paralogs
Source: PLoS One. 2016 Jan 4;11(1):e0146155. doi: 10.1371/journal.pone.0146155 (PMC4699748; doi:10.1371/journal.pone.0146155)

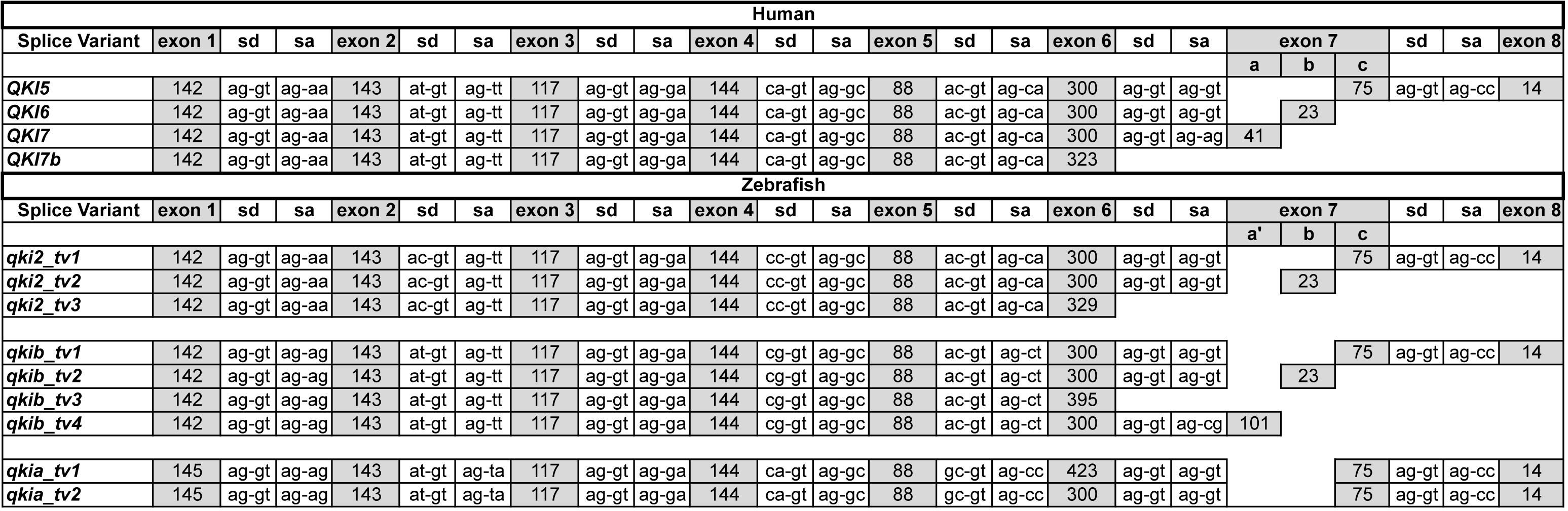

Supplement: S1 Fig — Known and predicted splice variants of the human QKI and the three zebrafish qki genes are detailed. The splice acceptor (sa) and splice donor (sd) sites for each exon and the exon length (in nucleotides) are compared between species. (TIF) [file pone.0146155.s001.tif]
